# In Vitro and In Vivo Anti-Inflammatory Activity of 17-*O*-Acetylacuminolide through the Inhibition of Cytokines, NF-κB Translocation and IKKβ Activity

**DOI:** 10.1371/journal.pone.0015105

**Published:** 2010-12-01

**Authors:** Mouna Achoui, David Appleton, Mahmood Ameen Abdulla, Khalijah Awang, Mustafa Ali Mohd, Mohd Rais Mustafa

**Affiliations:** 1 Department of Pharmacology, University of Malaya, Kuala Lumpur, Malaysia; 2 Department of Molecular Medicine, University of Malaya, Kuala Lumpur, Malaysia; 3 Department of Chemistry, University of Malaya, Kuala Lumpur, Malaysia; Universita di Sassari, Italy

## Abstract

**Background and Purpose:**

17-*O*-acetylacuminolide (AA), a diterpenoid labdane, was isolated for the first time from the plant species *Neouvaria foetida*. The anti-inflammatory effects of this compound were studied both in vitro and in vivo.

**Experimental Approach:**

Plant extracts were initially tested against LPS-stimulated release of tumor necrosis factor alpha (TNF-α) from murine macrophages (RAW264.7 cells). Based on bioassay-guided fractionation, the active compound was identified as AA. AA was tested for its ability to reduce nitric oxide (NO) production, and the inducible nitric oxide synthase (iNOS) expression. The inhibition of a panel of inflammatory cytokines (TNF, IL-1β, IL-6, KC, and GM-CSF) by AA was assessed at the expression and the mRNA levels. Moreover, the effect of AA on the translocation of the transcription factor nuclear factor kappa B (NF-κB) was evaluated in LPS-stimulated RAW264.7 cells and in TNF-stimulated L929 cells. Subsequently, AA was tested in the inhibitor of NF-κB kinase beta (IKKβ) activity assay. Lastly, the anti-inflammatory activity of AA in vivo was evaluated by testing TNF production in LPS-stimulated Balb/c mice.

**Key Results:**

AA effectively inhibited TNF-α release with an IC_50_ of 2.7 µg/mL. Moreover, AA significantly inhibited both NO production and iNOS expression. It significantly and dose-dependently inhibited TNF and IL-1β proteins and mRNA expression; as well as IL-6 and KC proteins. Additionally, AA prevented the translocation of NF-κB in both cell lines; suggesting that it is acting at a post receptor level. This was confirmed by AA's ability to inhibit IKKβ activity, a kinase responsible for activating NF-κB, hence providing an insight on AA's mechanism of action. Finally, AA significantly reduced TNF production in vivo.

**Conclusions and Implications:**

This study presents the potential utilization of this compound, as a lead for the development of an anti-inflammatory drug.

## Introduction

Inflammation, a healing response of the body to various insults, is a two edged sword. While it is typically recognized as a process for the remission of diseases, the persistence of this process may lead to various diseases associated with chronic inflammation [1], [2]; this includes arthritis, atherosclerosis, and even cancer [3], [4], [5]. Due to the resistance of such diseases to conventional treatments, as well as the side-effects of presently available anti-inflammatory drugs, there is a pressing need for the development of novel anti-inflammatory drugs. Natural products are a valuable source of novel bioactive secondary metabolites [6]. Various bioassays exist in which the anti-inflammatory activity of these products can be evaluated [7].

Macrophages have been implicated in many of these assays, as they are directly involved with the inflammatory response [8]. One of the important roles of macrophages is the production of various cytokines, reactive oxygen and nitrogen species, growth factors and chemokines as a response to activation signals such as chemical mediators, cytokines, and bacterial lipopolysaccharide (LPS) [9], [10]. Although the bioactive molecules produced by macrophages have valuable outcomes in inflammation, these molecules were also shown to have unfavorable and damaging effects [11]. Hence the modulation of these products provides a target for controlling inflammatory diseases. Of particular importance is the production of the cytokine tumor necrosis factor alpha (TNF-α) or (TNF).

**Figure 1:**
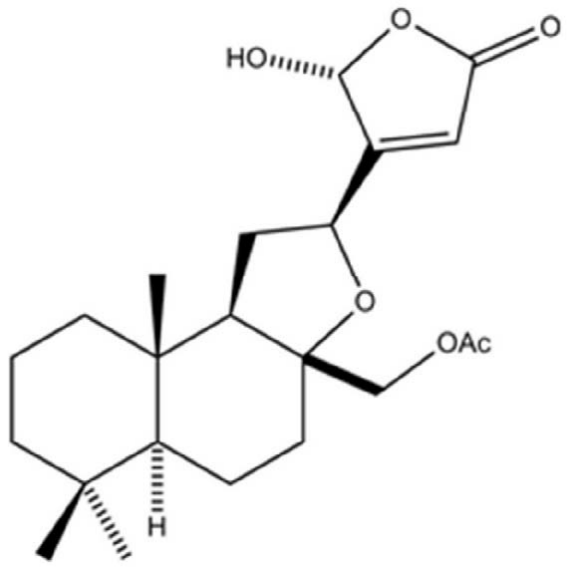
Chemical structure of 17-*O*-acetylacuminolide [(12S)-17-acetoxy-8a,12-epoxy-16(R)-hydroxylabd-13(14)Z-en-15,16-olide].

TNF was shown to affect various biological processes including the regulation and the production of other cytokines [12]. By utilizing an in vitro bioassay in which TNF production can be evaluated, compounds from natural products directed at inhibiting this cytokine can be identified. In this study, the screening of a library of Malaysian plants led to the isolation of the compound 17-*O*-Acetylacuminolide (AA) (Figure 1) from the plant *Neouvaria foetida*. The compound, a diterpene labdane, was shown to inhibit TNF production from murine macrophages. Terpenoids, a class of secondary metabolites under which 17-*O*-acetylacuminolide falls, are reported to have diverse biological activities [13]. The present study investigated the potential anti-inflammatory activity of 17-*O*-Acetylacuminolide in vitro and in vivo to evaluate its use as a possible lead compound.

## Methods

### Ethics statement

Animal experiments were performed in accordance with the guidelines for animal experimentation issued by the Animal Care and Use Committee at the University of Malaya (Ethics Number: FAR/11/03/2009/MA(R)).

### Cell lines and Reagents

RAW264.7 and L929 murine cell lines were obtained from American type culture collection (ATCC). Dulbecco's Modified Eagle Medium (DMEM) both with and without phenol red, phosphate buffered saline and Hanks' balanced salt solution (HBSS), 3-(4,5-Dimethylthiazol-2-yl)-2,5-diphenyltetrazolium bromide (MTT), Phosphate buffered saline (PBS) and Griess reagent were from Invitrogen (Carlsbad, USA). Fetal bovine serum (FBS), LPS from *E.coli* serotype 0111:B4, pentoxifillyne, aminoguanidine hydrochloride, curcumin, recombinant murine TNF-α, Dimethylsulfoxide (DMSO), and sodium nitrite were obtained from Sigma (St Louis, USA). Interferon gamma (IFNγ) was from BD Biosciences (New Jersey, USA). TNF-α ELISA kit was from eBioscience (San Diego, USA). Procarta cytokine assay and Quantigene plex 2.0 assay kits were from Panomics (Affymetrix) (Santa Clara, USA). NF-κB translocation and iNOS Activation kits were from Cellomics (Pittsburg, USA). K-LISA™ IKKβ-Inhibitor Screening Kit and dexamethasone were from Calbiochem, Merck (Darmstadt, Germany). All other chemicals and reagents used were of HPLC grade.

### Plant extraction

Plant material was obtained from the department of chemistry herbarium, University of Malaya, Malaysia. Dried leaves (500 g) of *Neouvaria foetida* (maingay ex Hk. f. et Th.) Airy shaw (specimen voucher number: KL5513), were extracted in methanol for 48 hours at room temperature with shaking. The solvent was dried by rotary evaporation under reduced pressure and at a maximal temperature of 40°C. The extract was then freeze-dried to yield crude methanol extract (45 g), which was stored at −20°C until further use.

### Chromatography and compound isolation

The crude extract was fractionated using reversed phase C_18_ flash column chromatography into seven fractions (A–G). After pre-conditioning with water, the crude extract was added to the column and eluted using a step gradient of water and methanol as follows: Fraction A: MeOH∶H_2_O (20∶80); B: MeOH∶H_2_O (30∶70); C: MeOH∶H_2_O (40∶60); D &E: MeOH∶H_2_O (50∶50); F: MeOH∶H_2_O (60∶40); G: MeOH∶H_2_O (100∶0). The fractions were analyzed using liquid chromatography mass spectrometry (LC-MS) (Shimadzu IT-TOF) with AA being detected largely in fraction G (8.4 g).

Fraction G was then subjected to preparative high performance liquid chromatography (HPLC) (Gilson GX-281/322 system) using a Waters Novapak C_18_ column (25×100 mm, 6 µm). The extract was eluted at a flow rate of 12 mLmin**^−1^** over 60 mins. The gradient started at 30% solvent (B) (acetonitrile with 0.1% formic acid) and 70% solvent A (water with 0.1% formic acid) for 5 mins, then 30–37% (B) for 5 min. The gradient then changed from 37% to 50% (B) over 45 minutes, followed by 50–100% (B) for 5 minutes. Finally, there was an isocratic elution of 100% (B) from 50 to 60 minutes. AA eluted at 36 min (Figure S1).

Final purification was achieved by re-crystallization from methanol to yield AA. Purity was determined using proton nuclear magnetic resonance (^1^H NMR) and Shimadzu LC-MS (Figures S2 and S3). For LC-MS analysis, the column used was BDS Hypersil (C_18_, 5 µm, 4.6×150 mm) and was maintained at 40°C, and the injection volume was 20 µl, sample concentration was 1 mgmL**^−1^**. The compound was eluted at a flow rate of 1 mLmin**^−1^** over 30 mins. Forty five percent solvent B (acetonitrile with 0.1% formic acid) flowed through the column with 55% solvent A (water with 0.1% formic acid) with a gradient of an increasing concentration of (B), reaching 75% (B) over 25 minutes. Solvent B concentration increased from 75–100% over one minute, then there was an isocratic elution with 100% solvent (B) from 26–30 minutes. The compound eluted at 10.35 minutes (Figure S3).

The identity and purity was also confirmed with ^1^H NMR using a Lambda 400 MHz FT-NMR spectrometer in CDCl_3_ and *d*_6_-dimethyl sulfoxide with all data being identical to that previously reported for AA [14]. AA mass and UV spectra are as depicted in (Figures S4, S5, S6). AA was dissolved in dimethyl sulfoxide (DMSO) and diluted in DMEM or PBS before it was used for testing its biological activity.

### Cell culture

Cells were maintained in high glucose DMEM with 10% FBS and no antibiotics, with passage every 2–3 days using standard aseptic techniques. Cells from 70–90% confluent flasks with >90% viability were seeded into 96-well culture plates at the densities indicated below.

### Cell viability and cytotoxicity

The viability of the cells was assessed using the MTT colorimetric assay as described in [15] with some modifications. RAW264.7 cells were either left untreated or were treated with AA at the indicated doses. Following 4 hours incubation, the medium was replaced with 100 µl fresh DMEM and 20 µl of 5 mgmL**^−1^** MTT and was further incubated for one hour. Subsequently, the cell medium was aspirated and 100 µl of 100% DMSO was added to all wells to dissolve the insoluble purple formazan product into a colored solution; the absorbance of which was measured at a wavelength of 570 nm using a microplate reader (Hidex Chameleon, Finland). The optical density (OD) of the samples was compared to that of the negative control to obtain the percentage viability as follows: Cell viability (%)  =  [(OD _570_ (sample)/OD_570_ (negative control)) ×100].

### TNF production

RAW 264.7 were seeded in 96-well plates at 5×10^5^cellsmL**^−1^** and pre-treated with AA or pentoxifilline at the indicated concentrations, or left untreated for 30 minutes. The cells were then stimulated with 1 µgmL**^−1^** LPS for 4 hours [16]. The cell supernatant was stored at −80°C until analysis. TNF quantification was done using mouse TNF-α ELISA kit according to the manufacturer's protocol (eBioscience, USA).

### Cytokines measurement

#### Multiplex cytokine assay

The Procarta cytokine profiling kit and the Quantigene Plex 2.0 reagent system for mRNA analysis [17], both from Panomics (Affymetrix, USA), were used to measure five cytokines in the mouse inflammatory panel: interleukin 1β (IL-1β), interleukin 6 (IL-6), Chemokine (C-X-C motif) ligand 1 (CXCL-1 or KC), granulocyte monocyte colony stimulating factor (GM-CSF), and TNF in the cell culture supernatants and cultured cells. Although IL-8 is an important proinflammatory chemokine in the human inflammatory response [18], this chemokine is not produced by murine cells. However, the cytokine KC (CXCL-1) which was tested in this study is a murine cytokine functionally homologous to the human IL-8 [19], [20]. The multiplex cytokine assay uses xMAP technology (multi-analyte profiling Luminex technology) to enable the detection and quantification of multiple protein targets simultaneously. RAW 264.7 were seeded in 96-well plates at 5×10^5^cellsmL**^−1^** and pre-treated with AA or pentoxifilline at the indicated concentrations, or left untreated for 30 minutes. The cells were then stimulated with 1 µgmL**^−1^** LPS for 4 hours. The cell supernatant and the cells were stored separately at −80°C until analysis. The samples were processed according to the manufacturer's protocol. For the protein quantification assays, MasterPlex QT4.0 software was used to analyze the concentration of each cytokine in each sample from the arbitrary unit median fluorescence intensity (MFI). The percentage inhibition was then calculated as follows:
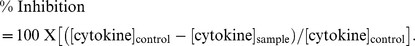
For the Quantigene Plex 2.0 assay, MFI was normalized to the house keeping gene GADPH, and the calculation of the fold-change percentage inhibition was calculated as the formula above, with the replacement of cytokine concentration values with the fold change in expression.

### Nitrite Determination and iNOS activation

#### Nitrite measurement

Tests were prepared as in [21] with some modifications. RAW 264.7 were seeded in 96-well plates at 5×10^5^cellsmL**^−1^** for 2 hours in DMEM without phenol red. The cells were stimulated with IFN-γ and LPS with final concentrations of 200 UmL**^−1^** and 10 µgmL**^−1^**, respectively. Stimulated cells were either treated with AA at different concentrations (1.25−0.125 µgmL**^−1^**) or were treated with the iNOS inhibitor aminoguanidine at 1 mM as a positive control; or were left untreated (negative control). The final volume per well was 100 µl. The plates were then incubated for 16–20 hrs at 37°C, 5% CO_2_. Following incubation, the inhibition of nitric oxide (NO) release was assessed by quantifying nitrite (NO_2_^−^), one of the final products of NO oxidation, in the cell supernatant by utilizing the Griess reaction [22]. The resulting color development was measured at 550 nm with a microplate reader Tecan Sunrise (Grödig, Austria). The absorbance values were compared to a standard sodium nitrite curve and the absorbance values were converted to corresponding nitrite concentrations (µM). The percentages of NO inhibition were calculated as follows:
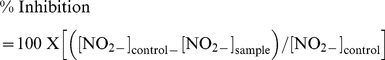



#### Inducible nitric oxide synthase (iNOS) activation assay

Murine RAW264.7 cells were seeded overnight in 96-well plates at a density of 2.5×10^5^ cells mL**^−1^**. The cells were pre-treated with either 1 mM aminoguanidine, different concentrations of AA or were left untreated, for 30 minutes. The cells were then stimulated with IFN-γ and LPS (200 UmL**^−1^** and 1 µgmL**^−1^**) for 18 hours, and the inhibition of iNOS induction in the cells was quantitatively assayed using Cellomics® iNOS activation kit according to the manufacturer's instructions. The assay plate was evaluated on ArrayScan HCS Reader instrument from Cellomics (Pittsburg, USA). The Cytoplasm to Nucleus Translocation BioApplication software was used to calculate the intensity of cytoplasmic iNOS intensity. The average intensity of 200 objects (cells) per well was quantified and percentage inhibition was calculated by comparing the various groups' cytoplasmic intensities to that of LPS and IFNγ stimulated cells.

### NF-κB Translocation assay

Murine RAW264.7 or L929 cells were seeded overnight at 1.2 or 2.5×10^5^ cellsmL**^−1^**; respectively, in a 96-well plate. The cells were either pre-treated for one hour with different concentrations of AA or were left untreated. RAW264.7 cells were then stimulated with 10 ngmL**^−1^** of LPS; whereas L929 cells were stimulated with 1 ngmL**^−1^** TNF, both for 30 minutes. The medium was discarded and cells were fixed and stained using Cellomics® NF-κB activation kit from Thermo Scientific according to the manufacturer's instructions. The assay plate was evaluated on ArrayScan HCS Reader. The Cytoplasm to Nucleus Translocation BioApplication software was used to calculate the ratio of cytoplasmic and nuclear NF-κB intensity [23]. The average intensity of 200 objects (cells) per well was quantified. The ratios were then compared among stimulated, treated, and untreated cells.

### Inhibitor of NF-κB Kinase beta (IKKβ) activity assay

The effect of AA on IKKβ activity was evaluated using K-LISA™ IKKβ-Inhibitor Screening Kit from Calbiochem, Merck (Darmstadt, Germany), according to the manufacturer's protocol. AA (3.1–50 µgmL**^−1^**) was incubated with the GST-IκBα substrate and IKKβ in the wells of a Glutathione-coated 96-well plate. The phosphorylated GST-IκBα substrate was detected using an Anti-Phospho IκBα (Ser32/Ser36) antibody, followed by the HRP-Conjugate and color development with TMB Substrate. ELISA Stop Solution was used to stop the color development and the absorbance was read at 450 nm. Absorbance was directly related to the level of IKKβ activity from which percentage inhibitions were calculated.

### Animals

Male Balb/c mice (5–6 weeks of age) were maintained under pathogen-free conditions in the animal housing unit in a temperature-controlled (23±2°C) and light-controlled (12-h light/dark cycle) room. The animals were provided standard rodent chow and water *ad libitum*.

### Measurement of in vivo serum TNF

The mice were divided into 4 groups: no treatment group, injected with phosphate buffered saline (PBS); LPS only; LPS+Dexamethasone (6 mgkg**^−1^**); or LPS+AA (100 mgkg**^−1^**) (n = 9–10 for each group). Groups were pretreated intraperitoneally (i.p.) with 200 µl of PBS, Dexamethasone or AA for 30 minutes. Lipopolysaccharide (1 mgkg**^−1^**) was then administered in 200 µl i.p for three treatment groups, and PBS was administered i.p. for the negative control group. Blood was withdrawn from the animals under ether anesthesia ninety minutes later [24]. Blood from mice was centrifuged at 2000 rpm for 10 minutes, and the serum was collected and stored at −80°C until analysis. Serum levels of TNF-α were determined using Mouse TNF-α ELISA kit (eBioscience, USA).

### Statistical Analyses

The assays were conducted in at least three separate experiments, unless otherwise specified. Data are expressed as the mean ± standard deviation. Data were analyzed using Graph pad prism statistical software (version 4; GraphPad Software Inc., La Jolla, California) for one-way analysis of variance (ANOVA) with Tukey's post hoc test, or Student's t-test as indicated. Differences were considered significant at *p*<0.05. The half maximal inhibitory concentration (IC_50_) was calculated using sigmoidal dose-response (variable slope) equation under non-linear regression (curve fit) utilizing the same software. Where inhibition is calculated; values from untreated, unstimulated cells were considered as the maximum inhibition (100%).

## Results

### 17-O-acetylacuminolide is a major component of *Neouvaria foetida*

*Neouvaria foetida* methanol extract exhibited effective inhibition of TNF release from LPS stimulated RAW264.7 macrophages in a preliminary screen of more than 300 Malaysian plant extracts. Bio-assay guided fractionation lead to the isolation of two active compounds which were identified as acuminolide and 17-*O*-acetylacuminolide by MS and ^1^H-NMR. Both compounds have previously been isolated from a different species of Neouvaria, namely *Neouvaria acuminatissima* (Miq) [14]. Upon recrystallization in methanol, 17-*O*-acetylacuminolide formed as colorless needles (98 mg, 0.0196% dry wt/wt, >98.5% purity by HPLC). Due to acuminolide's reported in vivo toxicity [14], the present study was confined to investigating the anti-inflammatory activities of AA, which even at 110 mg/kg did not seem to have any in vivo toxic effect [14].

### Cell viability and cytotoxicity

**Figure 2:**
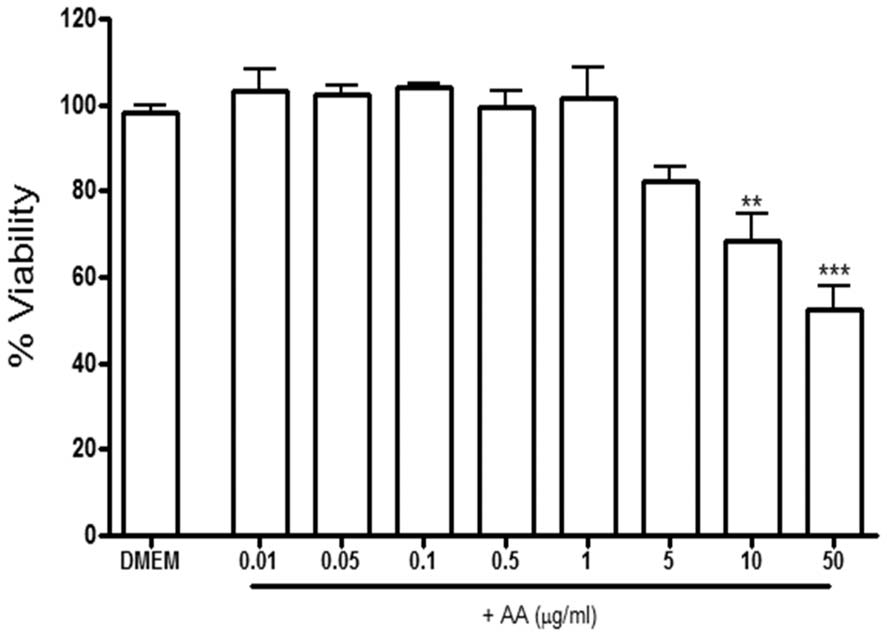
The effect of 17-*O*-acetylacuminolide (AA) on cell viability in RAW264.7 cells. Cells were pretreated with the indicated doses of AA for 4 hours or were left untreated (DMEM). Data is the average of three independent experiments (±SD), and was analyzed using one way ANOVA with Tukey's post test (** p<0.01, *** p<0.001).

The effect of AA on cell viability was evaluated using the MTT assay. As depicted in Figure 2, following a 4 h treatment, AA had no effect on RAW264.7 cell viability with concentrations ranging from 0.01–5 µgmL**^−1^**. However, the highest dose tested (50 µgmL**^−1^**) resulted in a 50% decrease in viability. Therefore for prolonged assays (>4 h), the pharmacological effects of this compound were assessed at lower, non-cytotoxic concentrations. An exception was the NF-κB translocation assay where high doses were used since the assay duration was short, and no significant toxicity was observed up to 40 µgmL**^−1^**.

### 17-O-Acetylacuminolide inhibits TNF production

**Figure 3:**
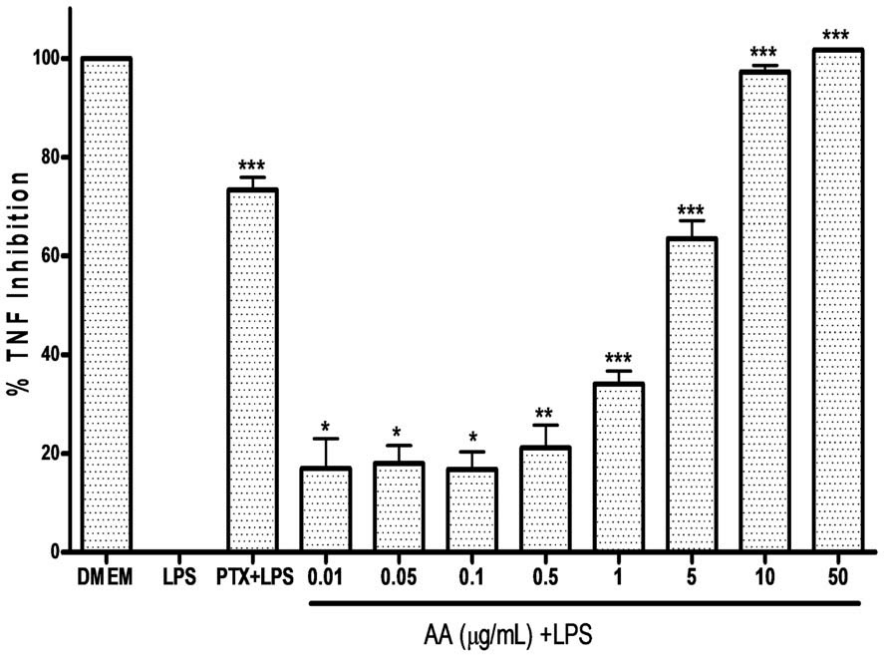
The inhibitory effect of 17-*O*-acetylacuminolide (AA) on TNF production in RAW264.7 cells. Cells were pretreated with the indicated doses of AA, or the TNF inhibitor pentoxifylline (PTX). The cells were stimulated with LPS (1 µgmL**^−1^**) for four hours, or were left untreated (DMEM).The protein concentration was measured using ELISA. Data is representative of three independent experiments, and was analyzed using one way ANOVA with Tukey's post hoc test (** p<0.01, *** p<0.001).

Stimulation of RAW264.7 with LPS for 4 hours caused a significant increase in TNF production. In the enzyme-linked immunoassay, AA effectively inhibited TNF-α release with an IC_50_ of 2.7 µgmL**^−1^**. As shown in Figure 3, AA dose-dependently and significantly suppressed TNF production in RAW264.7 cell supernatant at non-cytotoxic doses ranging from 5–0.01 µgmL**^−1^**.

### Effects of 17-O-Acetylacuminolide on inflammatory cytokines production and expression

LPS is known to activate the transcription process of various inflammatory mediators and cytokines [11]. Table 1 presents a summary of the effects of LPS stimulation in RAW264.7 cells. There was a significant release of all five cytokines in the supernatant. Also, there was a significant production of TNF, IL-1β, IL-6 and KC but not GM-CSF in the cell lysates (intracellular protein). At the transcription level, four hours of LPS treatment at 1 µgmL**^−1^** significantly induced the transcription of TNF and IL-1β mRNA, but not IL-6, CXCL-1 (KC), or CSF-2 (GM-CSF).

**Table 1:** Comparing protein concentrations and gene expression of 5 pro-inflammatory cytokines in untreated cells and stimulated cells.

	Sample	Protein Conc. Supernatant (pgmL^−1^) (±STDEV)	P value	Protein Conc. Lysate (pgmL^−1^) (±STDEV)	P value	Normalized mRNA (fold-change) (±STDEV)	P value
**TNF**	Stim.	2510.71 (±708.32)	**	122.31 (±41.03)	**	41.54 (±13.64)	**
	DMEM	17.17 (±3.88)		2.01 (±0.32)		2.14 (±0.57)	
**IL-1β**	Stim.	212.06 (±28.49)	***	21.28 (±6.22)	**	39.80 (±14.57)	**
	DMEM	6.68 (±4.73)		3.08 (±0.00)		0.15 (±0.08)	
**IL-6**	Stim.	60.64 (±4.37)	***	0.75 (±0.18)	*	0.61 (±0.39)	ns
	DMEM	1.22 (±0.00)		0.06 (±0.00)		0.25 (±0.19)	
**KC/CXCL-1**	Stim.	21.81 (±3.17)	***	1.43 (±0.43)	**	46.82 (±26.00)	ns
	DMEM	1.22 (±0.00)		0.25 (±0.00)		28.07 (±22.99)	
**GMCSF/CSF-2**	Stim.	38.46 (±4.49)	***	5.16 (±0.39)	Ns	25.07 (±13.43)	ns
	DMEM	1.42 (±0.35)		4.41 (±0.99)		13.80 (±11.14)	

Cells were either left untreated in medium (DMEM), or were stimulated with 1 µgmL**^−1^** LPS (Stim.) for four hours (stimulated). The table provides a comparison in protein concentrations (Protein Conc.) in the cell supernatant and lysates, as well as differences in gene expression. Student's t-test was used to calculate the statistical significance between the two groups (ns: not significant, * p<0.05, ** p<0.01, *** p<0.001).

**Figure 4:**
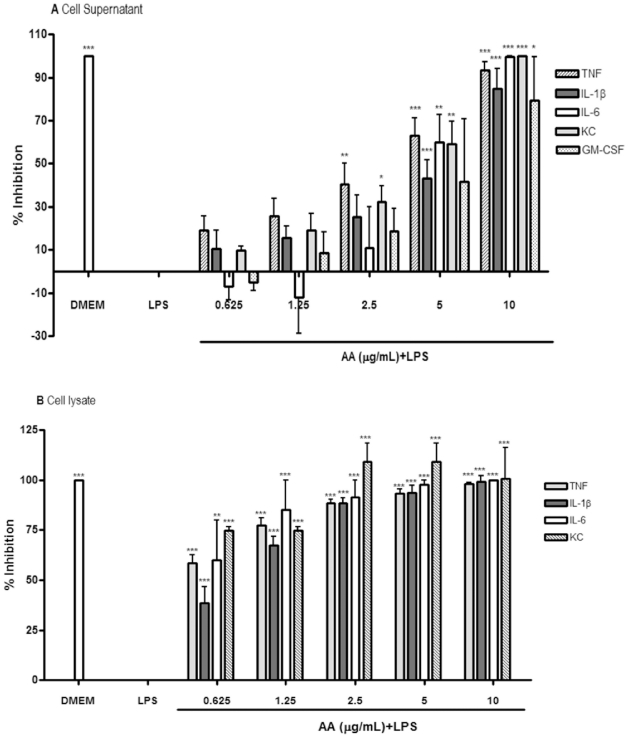
The inhibitory effect of 17-*O*-acetylacuminolide (AA) on cytokine release and synthesis in RAW264.7 cells. Cells were pretreated with the indicated doses of AA. The cells were stimulated with LPS (1 µgmL^−1^) for 4 hrs, or were left untreated (DMEM). Supernatant (A) and intracellular (B) protein concentrations were measured using Procarta 5-plex cytokine profiling kit from which inhibitions were calculated. Data is representative of three independent experiments, and was analyzed using one-way ANOVA with Tukey's post hoc test (* p<0.05, ** p<0.01, *** p<0.001).

In this multiplex assay, AA was tested for its ability to inhibit TNF, IL-1β, IL-6, KC (CXCL-1), and GM-CSF both at the protein production level and at the transcription level. Figure 4 (A) depicts the percentage inhibition of cytokines released into the supernatant of cell samples pretreated with AA as compared to cells treated with LPS alone (LPS). AA demonstrated a dose-dependent pattern of inhibition for almost all cytokines tested. Moreover, AA significantly and dose-dependently reduced intracellular increase of four pro-inflammatory cytokines in LPS treated samples as seen in Figure 4 (B). However, AA did not affect GM-CSF concentrations, presumably because there was no difference in this cytokine's concentration between LPS-stimulated and untreated cells (Table 1), this is in accordance with a previous report in which GM-CSF concentrations of cell lysates was less than 40% that of the corresponding supernatants [25].

**Figure 5:**
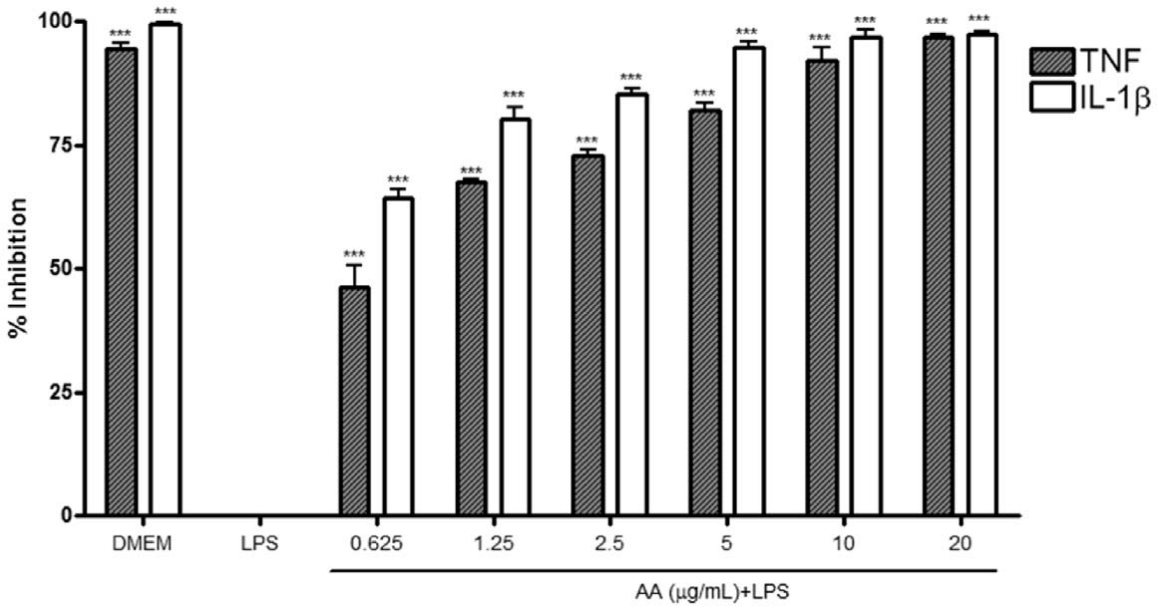
The inhibitory effect of 17-*O*-acetylacuminolide (AA) on cytokine mRNA. Cells were treated and mRNA was quantified as described in materials and methods. Data presented is of AA inhibition of TNF and IL-1β mRNA induction and is representative of three independent experiments. Data was analyzed using one-way ANOVA with Tukey's post hoc test (*** p<0.001).

At the transcription level (Figure 5), AA seemed to inhibit TNF and IL-1β, both of which were up-regulated significantly by LPS stimulation. The compound did not seem to affect the transcription of the 3 other cytokines tested. Although LPS stimulation seemed to increase the mRNA for IL-6, CXCL-1 and CSF-2; the mRNA levels for these 3 genes were not significantly different from the untreated cells (Table 1).

### Nitrite Determination and iNOS activation

**Figure 6:**
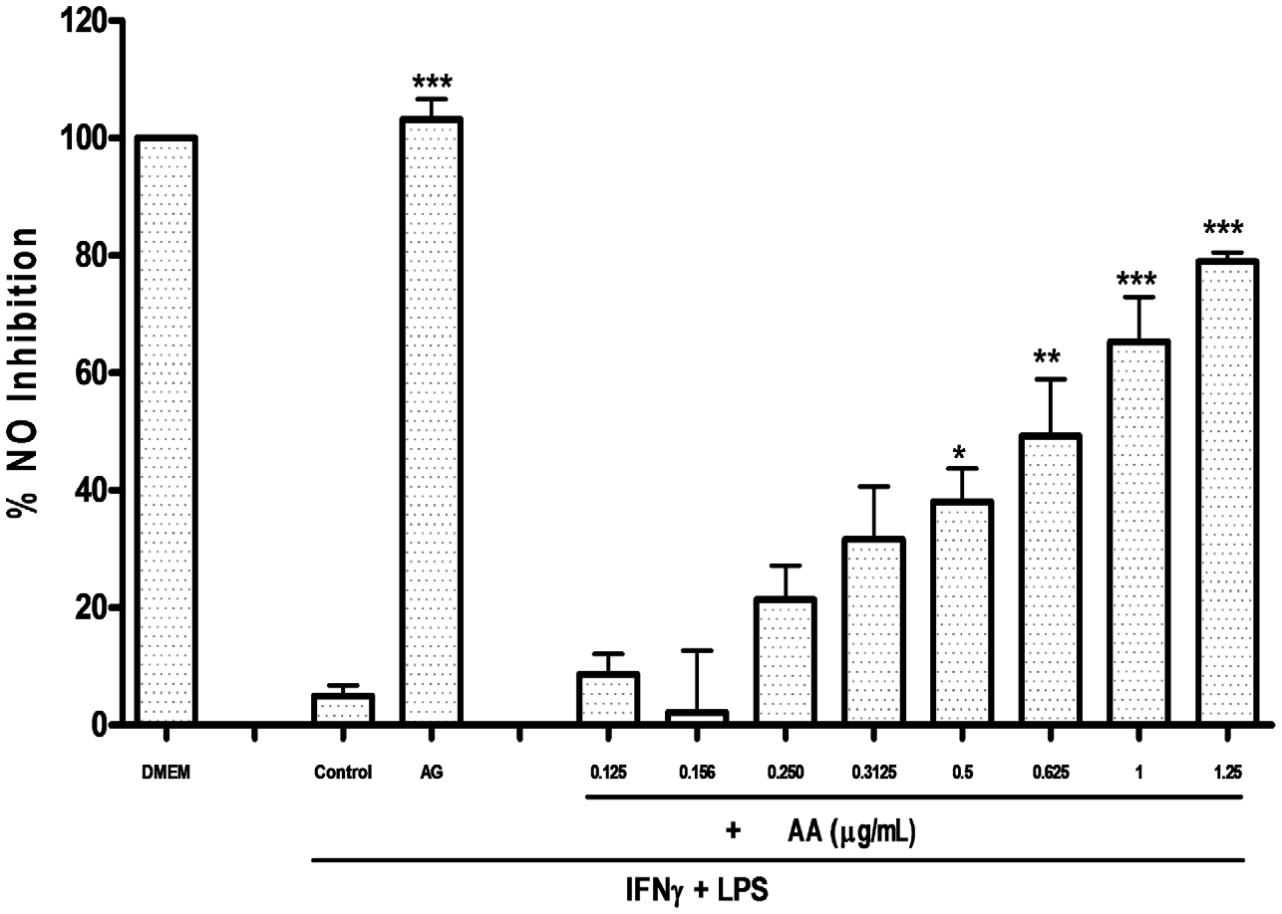
Percentage inhibition of nitric oxide (NO) in RAW264.7. Cells were pretreated with the indicated doses of 17-*O*-acetylacuminolide (AA) or with 1 mM of the iNOS inhibitor Aminoguanidine (AG). The cells were stimulated with LPS and IFNγ to activate iNOS synthesis, and eventually NO release. Data presented is the percentage of NO inhibition compared to untreated (DMEM), stimulated cells (control). Data is the average of three independent experiments (±SD), and was analyzed using one way ANOVA with Tukey's post test (* p<0.05, ** p<0.01, *** p<0.001).

Stimulation with LPS and IFN-γ led to a fifty six-fold increase in nitrite concentrations in the cell supernatant with a concentration of 157 µM as compared to the basal level of 2.75 µM in untreated cells. NO release was effectively inhibited by AA with an IC_50_ of 0.62 µgmL**^−1^**. Following 20 hours of treatment and stimulation, AA caused a 78.9% inhibition of NO at 1.25 µgmL**^−1^**, without affecting the cell viability (Figure 6).

**Figure 7:**
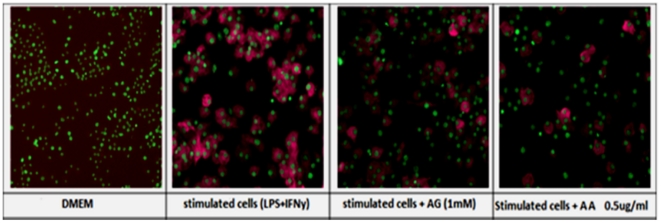
Effect of AA on iNOS expression in RAW264.7 cells. In untreated cells, the nuclei of the cells appear green (hoescht) and iNOS expression was undetected (DMEM). iNOS (DyLight™ 488, magenta), was expressed in the cell cytoplasm of stimulated cells. Pretreatment with aminoguanidine (AG) or 17-*O*-acetylacuminolide (AA) was able to inhibit iNOS expression in cells stimulated with lipopolysaccharide and interferon gamma (LPS+IFNγ).

**Figure 8:**
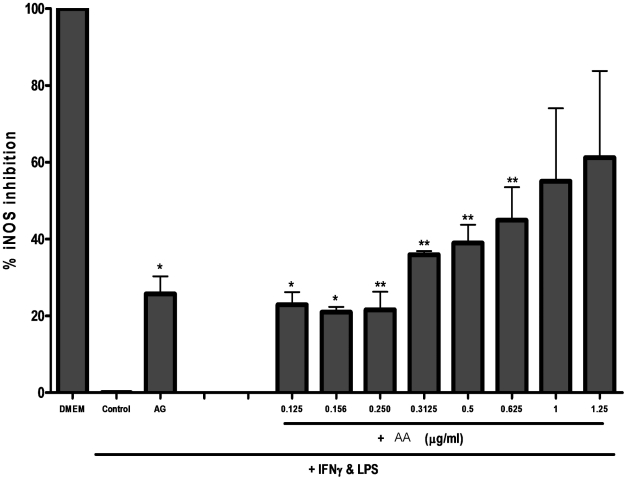
Percentage inhibition of inducible nitric oxide synthase (iNOS) in RAW264.7. Cells were pretreated with the indicated doses of AA or with 1 mM of the iNOS inhibitor Aminoguanidine (AG). The cells were stimulated with LPS and IFNγ to activate iNOS. Data presented is the percentage of iNOS inhibition compared to untreated, stimulated cells (control), and is the average of two experiments. Data was analyzed using one way ANOVA and Tukey's post hoc analysis (* p<0.05, ** p<0.01). Cell count was unaffected at the doses tested (data not shown).

Treatment of RAW264.7 cells with LPS and IFNγ lead to a 2.45-fold increase in iNOS protein expression in the cytoplasm. The increase in intensity of this inducible protein in the cytoplasm is directly related to its expression in the cell (Figure 7). AA suppressed the up-regulation of the inducible form of the nitric oxide synthase enzyme (iNOS). The same pattern of NO inhibition was observed in that for iNOS inhibition by AA. Percentage inhibition was calculated by comparing the change in average intensity between groups of cells, where LPS and IFN-treated group of cells was considered as zero percent inhibition, (control) in Figure 8. At 1.25 µgmL**^−1^**, AA caused ∼60% inhibition of iNOS expression whereas the lowest concentration of AA (0.126 µgmL**^−1^**) tested conferred an almost similar inhibition as that of the iNOS inhibitor aminoguanidine (AG); 22% and 25% inhibition, respectively. The calculated EC_50_ for AA iNOS inhibition was estimated at 0.96 µgmL**^−1^**.

### 17-O-acetylacuminolide inhibits NF-κB Translocation

**Figure 9:**
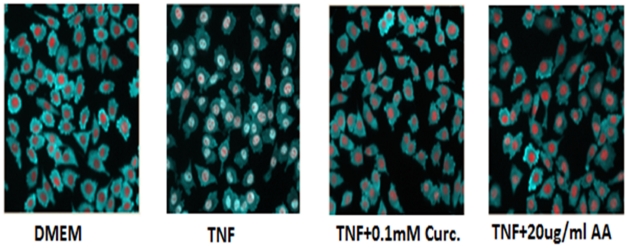
Effect of AA on TNF-stimulated L929 cells. NF-κB (Dylight™488, light blue); was sequestered in the cytoplasm of cells treated with media only (DMEM), the nuclei of the cells appear red (Hoescht). Upon TNF stimulation (TNF), NF-κB translocated to the nucleus. Pretreatment with curcumin (curc.), or 17-*O*-acetylacuminolide (AA) was able to prevent NF-κB translocation in the presence of TNF. (AA IC_50_ = 10.9 µgmL^−1^).

**Figure 10:**
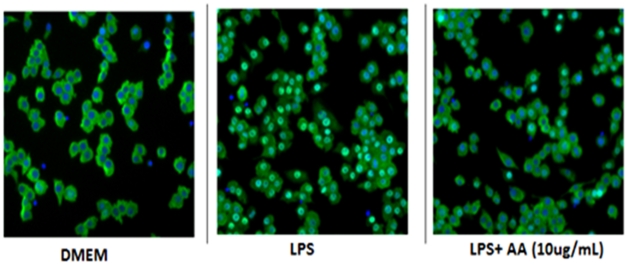
Effect of AA on LPS-stimulated RAW264.7 cells. NF-κB (DyLight™ 488, light green), was sequestered in the cytoplasm in cells treated with DMEM alone (untreated), and the nuclei of the cells appear blue (Hoescht). However, NF-κB translocates into the nucleus upon LPS stimulation. Pretreatment with 17-*O*-acetylacuminolide (AA) was able to prevent NF-κB translocation in the presence of LPS (IC_50_ = 7.8 µgmL**^−1^**).

**Figure 11:**
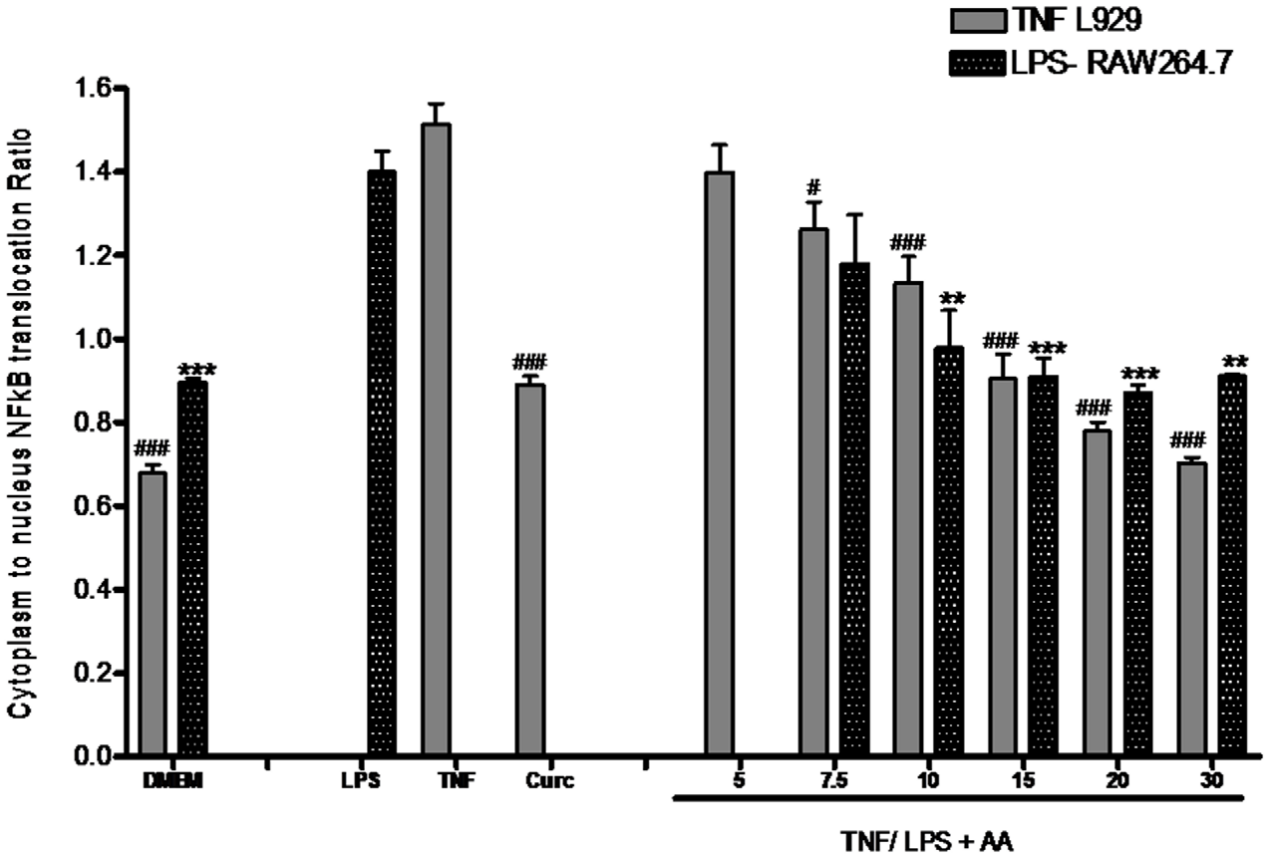
The inhibitory effect of 17-*O*-acetylacuminolide (AA) on the increase of nuclear NF-κB intensity. Cells were pretreated with the indicated doses of AA or with 0.1 mM of the NF-κB inhibitor Curcumin (Curc). L929 cells were stimulated with TNF (1 ngmL^−1^), whereas RAW264.7 cells were stimulated with LPS (10 ngml^−1^) to cause NF-κB translocation to the nucleus, or were left untreated (DMEM). Data is the average of three independent experiments (±SD), and was analyzed using one way ANOVA with Tukey's post test. The effect was considered significant when groups were compared to TNF (#) or LPS (*) treated L929 and RAW264.7 cells, respectively. (# p<0.05, ** p<0.01, ###, *** p<0.001).

The ability of AA to inhibit the transcription factor NF-κB was investigated. Treatment of L929 fibroblasts with TNF (1 ngmL**^−1^**) for 30 minutes lead to a 2.2-fold increase in the cytoplasm:nucleus NF-κB ratio (Figure 9). Whereas treatment of RAW264.7 cells with 10 ngmL**^−1^** LPS for 30 minutes lead to a 1.6-fold increase as compared to untreated cells (Figure 10). Pretreatment of stimulated cells with AA inhibited the translocation of NF-κB to the nucleus in both TNF-stimulated and LPS-stimulated cells in a dose-dependent manner (Figure 11).

### 17-O-Acetylacuminolide inhibits IKKβ activity

**Figure 12:**
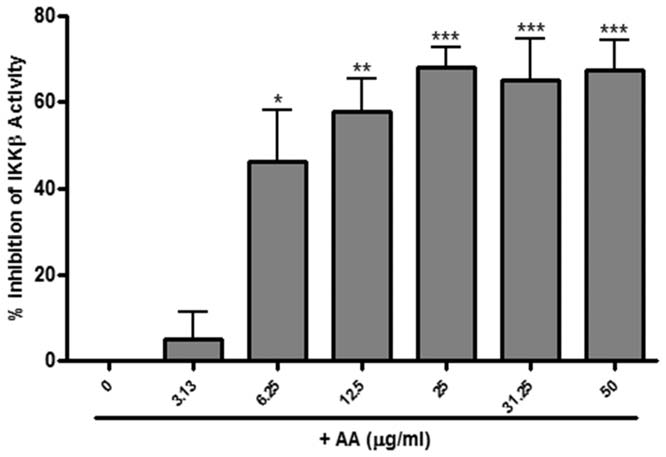
Effects of 17-*O*-acetylacuminolide (AA) on IKKβ activity. Human, recombinant IKKβ (5 ng) was incubated in the presence of increasing concentrations of AA as outlined in the manufacturer's detailed protocol. AA dose-dependently inhibited IKKβ activity with an EC_50_ of 5.2 µgmL**^−1^**. Results are average of three independent experiments ±SD. Data was analyzed using one-way ANOVA, with Tukey's post hoc test (* p<0.05, ** p<0.01, *** p<0.001).

As can be seen in Figure 12, AA significantly inhibited the activity of IKKβ, with a maximum inhibition of 68% at the highest concentration tested (50 µgmL^−1^).

### 17-O-Acetylacuminolide decreases serum TNF in LPS-stimulated Balb/c mice

**Figure 13:**
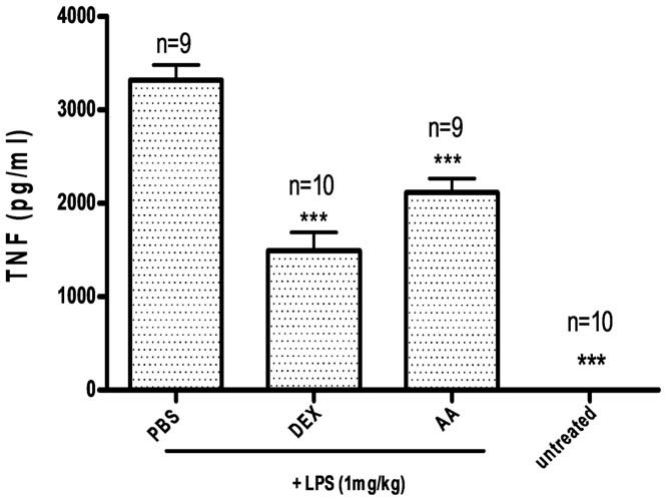
Effect of 17-O-acetylacuminolide (AA) on serum TNF levels in mice. Mice were pretreated i.p. with either 100 mgkg**^−1^** of AA, 6 mgkg**^−1^** of dexamethasone (DEX) or with Phosphate buffer saline (PBS) and DMSO (untreated) for 30 mins. The mice were then either injected with 1 mgkg**^−1^** lipopolysaccharide (LPS) or with PBS for 90 mins. Blood was withdrawn, and serum TNF was quantified using ELISA. One way ANOVA with Tukey's post analysis was used to calculate the statistical significance among the groups when compared to PBS+LPS group; *** p<0.001.

Injecting mice with LPS lead to >3000-fold increase in serum TNF levels compared to untreated mice. AA significantly reduced the serum TNF level by 35% in the LPS-stimulated animals (Figure 13). Pretreatment with the anti-inflammatory steroid dexamethasone caused a 54% reduction in serum TNF in LPS-stimulated mice.

## Discussion

In an effort to search for novel anti-inflammatory agents from Malaysian plants, more than 300 plant extracts were tested for their ability to reduce TNF production from LPS-stimulated RAW264.7 macrophages. The methanol extract of *Neouvaria foetida* leaves exhibited effective inhibition of TNF release; this consequently led to the identification of two active compounds acuminolide and 17-*O*-acetylacuminolide as being responsible for the observed activity. Although both acuminolide and 17-*O*-acetylacuminolide were isolated previously [14], to the best of our knowledge this is the first time their isolation from the species *Neouvaria foetida* is reported. Moreover, this is the first time the compounds were shown to reduce TNF production. TNF-α plays a pivotal role in several inflammatory conditions [26]. In order to evaluate the compound's activity, we further examined the anti-inflammatory effects of this compound on other inflammatory cytokines and mediators. TNF, IL1-β, IL-6, KC (CXCL-1) and GM-CSF were all shown to be up-regulated by LPS treatment [12], [27], [28], [29], [30]. Amongst the cytokines notably produced by LPS stimulation in this study, AA significantly reduced the production of TNF and IL-1β at both the transcription and translation levels. Upon stimulation with LPS, the various inflammatory mediators such as cytokines, NO and chemokines are induced at varying magnitudes and at different time points in response to the same stimulus [12], [17], [31]. This could explain, at least in part, the reason for lack of a significant difference in some of the cytokine's mRNA fold change between untreated and LPS-stimulated cells, as is the case for IL-6, CXCL-1 and CSF-2 (Table 1), and hence the reason for the lack of capacity to assess AA's effect on these cytokine genes.

Although AA significantly and dose-dependently reduced TNF production with a concentration as low as 10 ngmL**^−1^** in the ELISA assay, the inhibition was not significant for the lowest dose (6.25 µgmL**^−1^**) tested in the Procarta multiplex cytokine assay. However, the pattern of inhibition was still present in the latter assay. This may be due to the different methods used [32], as the Procarta cytokines assay is based on multiplexed measurement of fluorescence beads, whereas the ELISA method is a solid phase and TNF specific assay. It had been reported that using kits from various manufacturers for cytokines measurement yielded similar patterns, but different absolute concentrations [33].

Nitric oxide plays an important role in various inflammatory conditions where it is produced by the inducible form of nitric oxide synthase (iNOS) [34]. LPS and IFN-γ were shown to induce the expression of this enzyme, resulting in the production of abundant amounts of NO [35]. In addition to down-regulating several inflammatory cytokines, AA dose-dependently inhibited the production of nitric oxide. Moreover, the compound seemed to inhibit the expression of the enzyme responsible for the production of this inflammatory mediator (figures 7 and 8).

The inducible transcription factor NF-kB is responsible for the regulation of a plethora of genes involved in carcinogenesis and inflammation [4]. Due to the fact that the production of both iNOS [36] and pro-inflammatory cytokines and mediators [37] are regulated, at least in part, by the transcription factor NF-κB, we investigated the role AA plays on inhibiting this transcription factor's translocation. LPS as well as TNF were shown to cause NF-κB activation in RAW264.7 [38] and L929 cells [39], respectively. AA was able to inhibit the translocation of NF-κB in both LPS-treated RAW264.7 cells and TNF-treated L929 cells. This suggests that AA acts via a post-receptor signaling mechanism, since the pro-inflammatory stimulators used, i.e. LPS and TNF, act on different receptors, Toll like receptor 4 (TLR4) [40] and TNF receptor [41], respectively. The activation of NF-κB converges at the inhibitor of kappa B kinase (IKK) complex [42]. In the cell cytoplasm, NF-κB is sequestered by the inhibitor of kappa B (IκBα) protein [43]. The IKK complex phosphorylates IκBα protein upon activation by pro-inflammatory stimulators (e.g. TNF, LPS, IL-1β) [44]. This in turn leads to the activation of NF-κB and its translocation to the nucleus [45]. AA significantly inhibited the activity of IKKβ, which, along with IKKα comprise the catalytic part of the IKK complex [46]. Therefore, the inhibition of the IKK complex points to a possible mechanism of action by which AA inhibits NF-κB translocation and exerts its anti-inflammatory effects.

It was shown previously [14] that AA had potential in vitro activity against prostate (LNCaP) and melanoma (Mel2) cancer cell lines. The inactivation of NF-κB activity in these cell lines was shown to cause apoptosis [47] and [48]. Since AA was shown to inhibit NF-κB translocation, the cytotoxic effects of this compound may be attributed, to a certain extent, to the inhibition of this transcription factor.

Having demonstrated that AA possesses anti-inflammatory activity in vitro, we next tested it in an in vivo model of inflammation. In this model of in vivo acute inflammation [24], TNF release in mice serum dropped markedly upon pretreatment with AA. It has been demonstrated that several natural product compounds which fall under the class of terpenoids act as strong inhibitors of NF-κB activation [13]. Bearing in mind that 17-*O*-acetylacuminolide is a diterpene labdane, it is no surprise that this compound possesses potent anti-inflammatory activity as described in this study, as well as cytotoxic activity as described in [14]. This summarizes the potential value of 17-*O*-acetylacuminolide as a lead compound for the development of an anti-cancer or an anti-inflammatory drug, and warrants further testing of this compound in human cells models of inflammation.

## Supporting Information

Figure S1 **Chromatogram of** ***Neouvaria foetida*** **methanolic extract.** HPLC chromatogram of the crude extract, arrows indicate the peaks of acuminolide (**1**) and 17-*O*-acetylacuminolide (**2**).
(TIF)

Figure S2 **^1^H NMR spectrum of 17-** ***O*** **-acetylacuminolide (2) in CDCl_3_.**
(TIF)

Figure S3 **TIC mass chromatogram of 17-** ***O*** **-acetylacuminolide (2).**
(TIF)

Figure S4 **Mass spectra of 17-** ***O*** **-acetylacuminolide (2).** The spectra are a result of negative ionization mass spectrometry (MS^−1^) and tandem spectrometry (MS/MS)
(TIF)

Figure S5 **UV chromatogram of 17-** ***O*** **-acetylacuminolide (2).**
(TIF)

Figure S6 **Extracted UV Spectrum of 17-** ***O*** **-acetylacuminolide (2).**
(TIF)
