# Supplementary figures and images for: In Vitro and In Vivo Anti-Inflammatory Activity of 17-O-Acetylacuminolide through the Inhibition of Cytokines, NF-κB Translocation and IKKβ Activity
Source: PLoS One. 2010 Dec 1;5(12):e15105. doi: 10.1371/journal.pone.0015105 (PMC2995738; doi:10.1371/journal.pone.0015105)

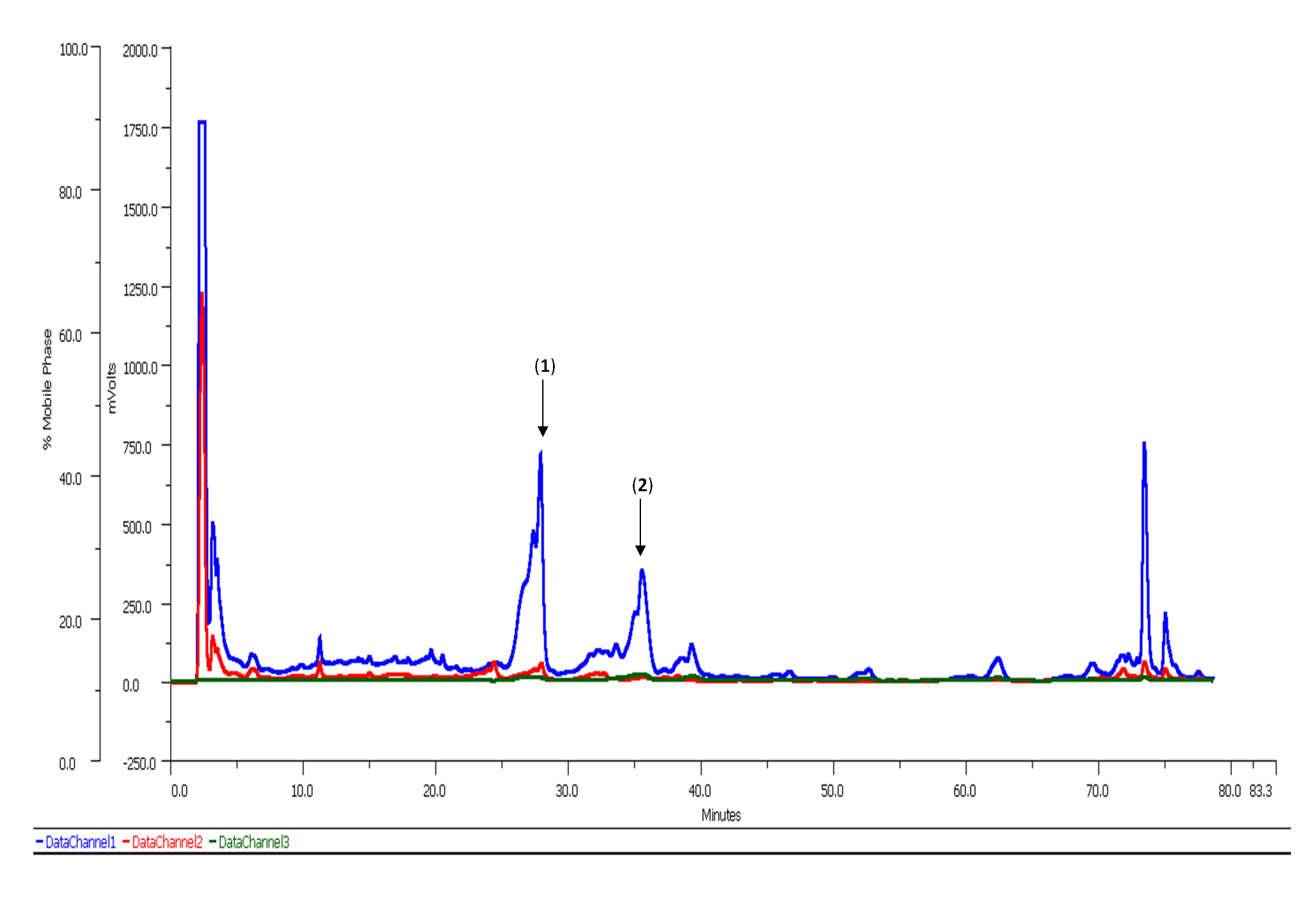

Supplement: Figure S1 — Chromatogram of Neouvaria foetida methanolic extract. HPLC chromatogram of the crude extract, arrows indicate the peaks of acuminolide (1) and 17-O-acetylacuminolide (2). (TIF) [file pone.0015105.s001.tif]

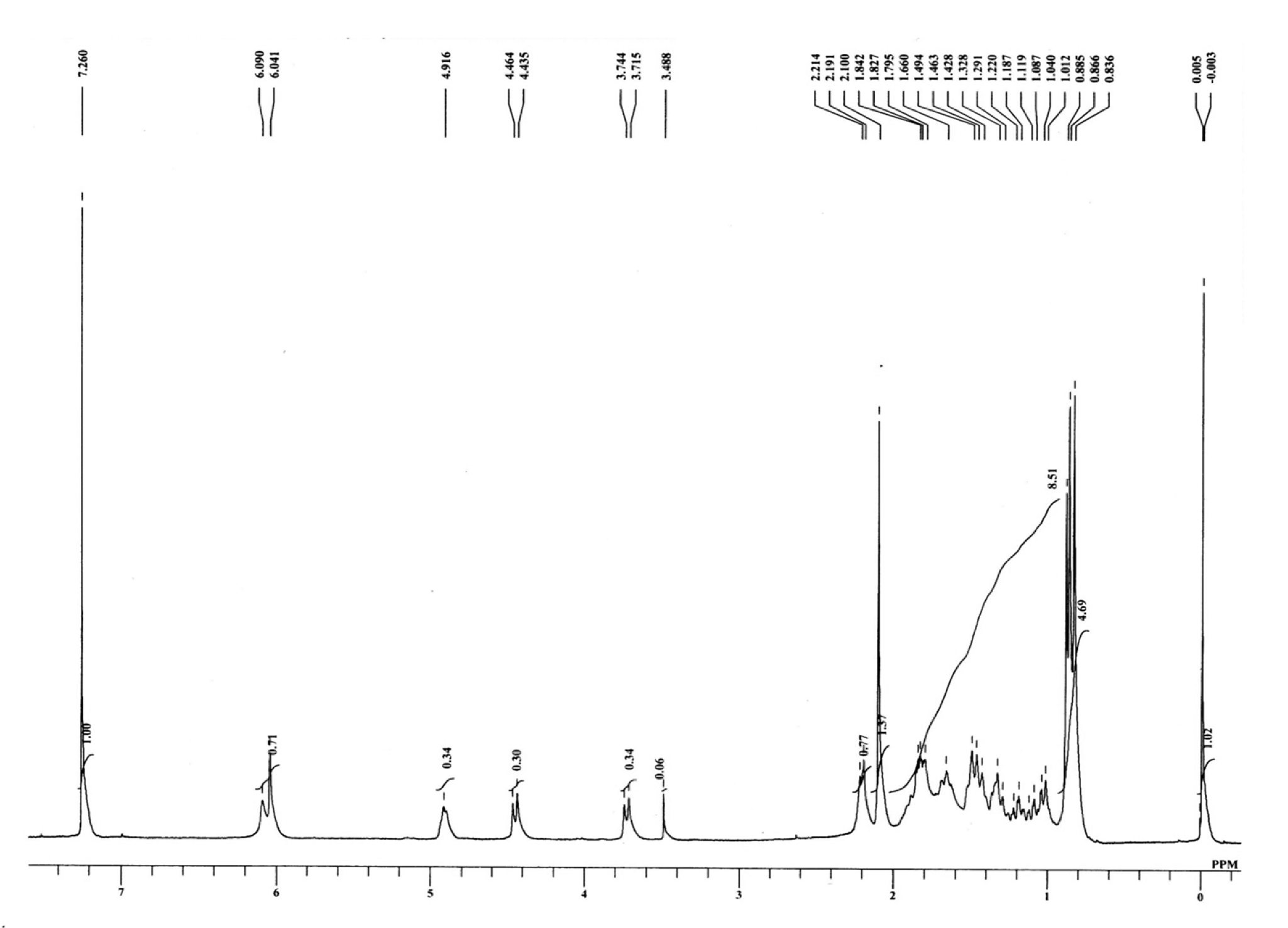

Supplement: Figure S2 — 1H NMR spectrum of 17- O -acetylacuminolide (2) in CDCl3. (TIF) [file pone.0015105.s002.tif]

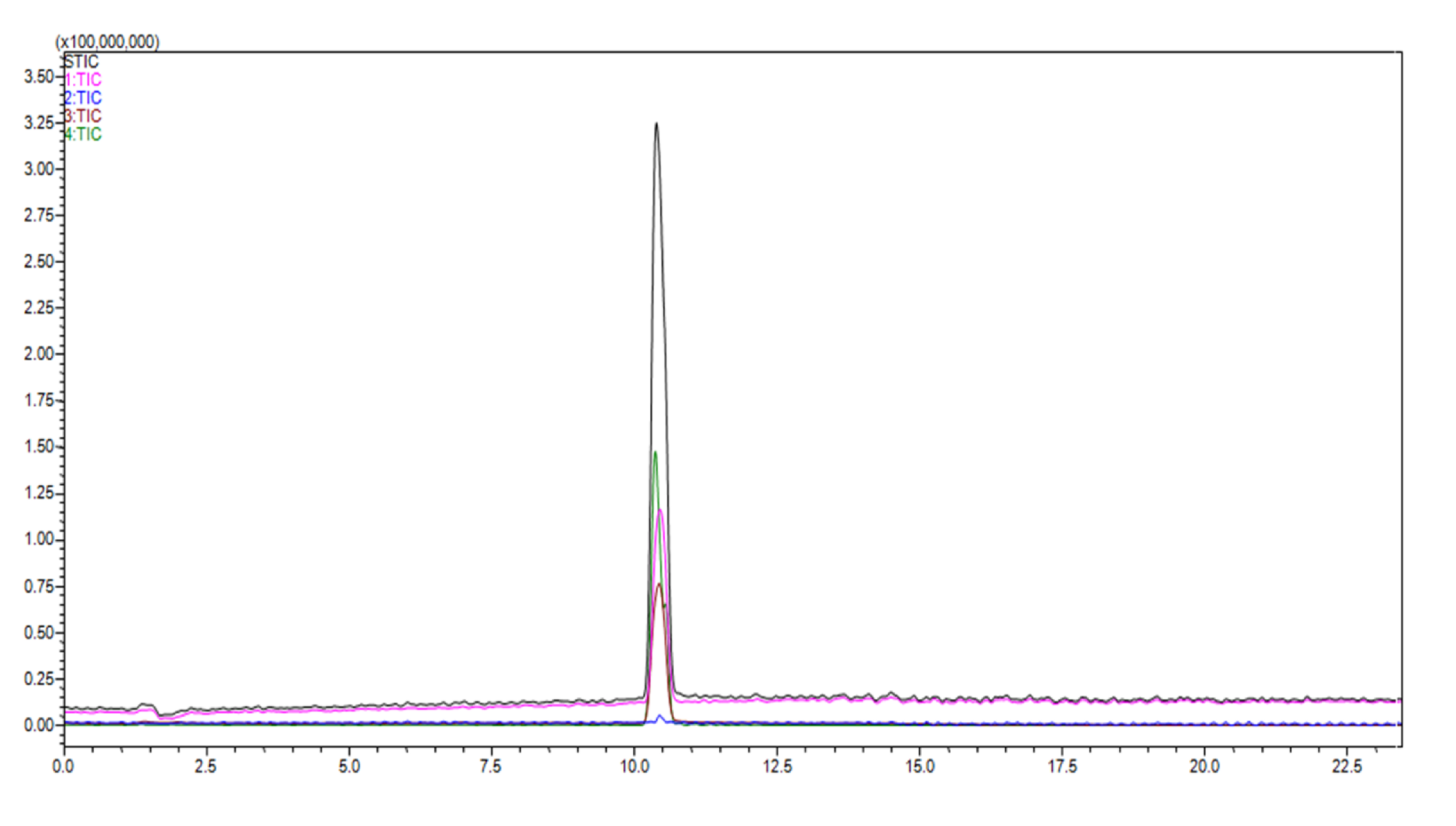

Supplement: Figure S3 — TIC mass chromatogram of 17- O -acetylacuminolide (2). (TIF) [file pone.0015105.s003.tif]

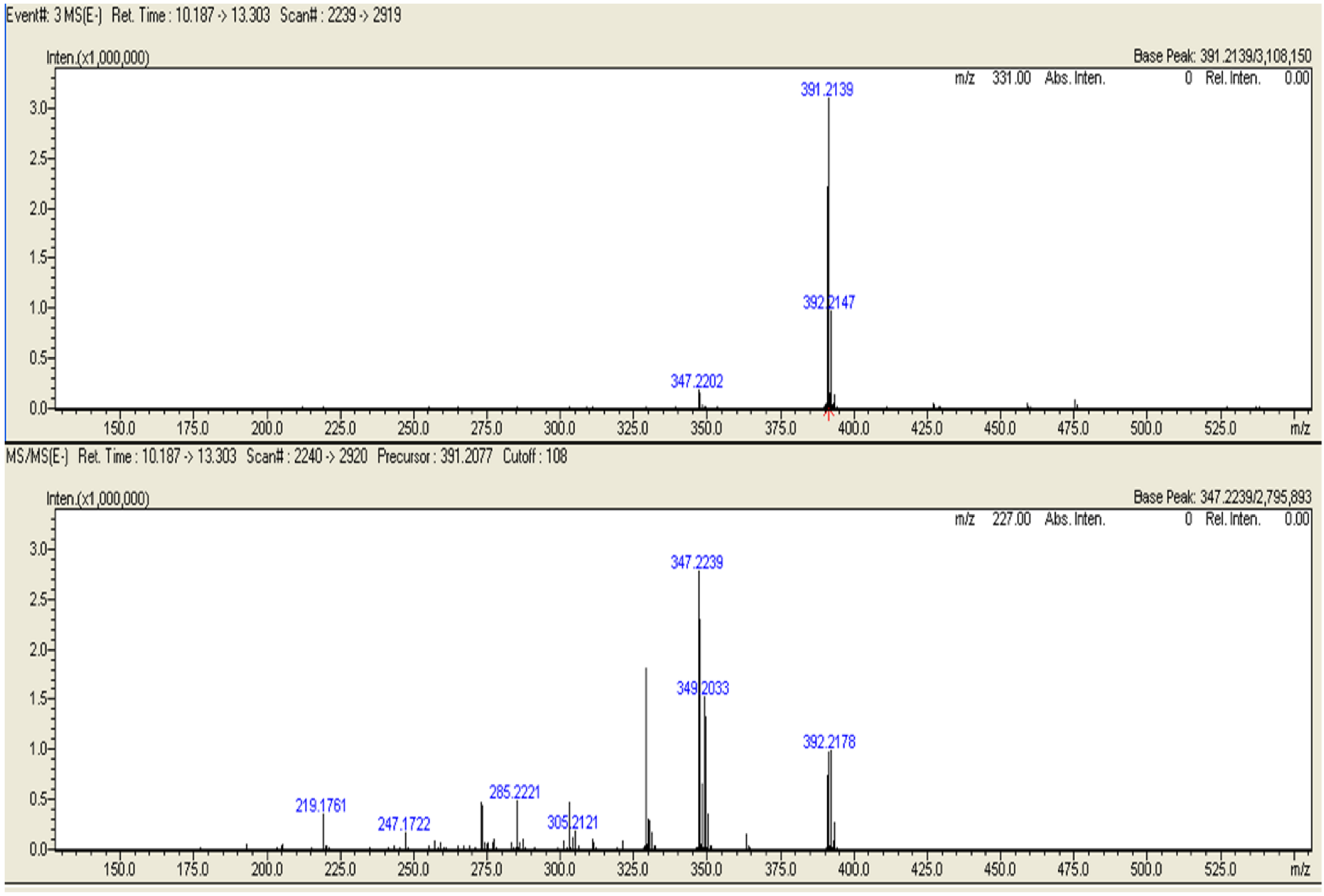

Supplement: Figure S4 — Mass spectra of 17- O -acetylacuminolide (2). The spectra are a result of negative ionization mass spectrometry (MS−1) and tandem spectrometry (MS/MS) (TIF) [file pone.0015105.s004.tif]

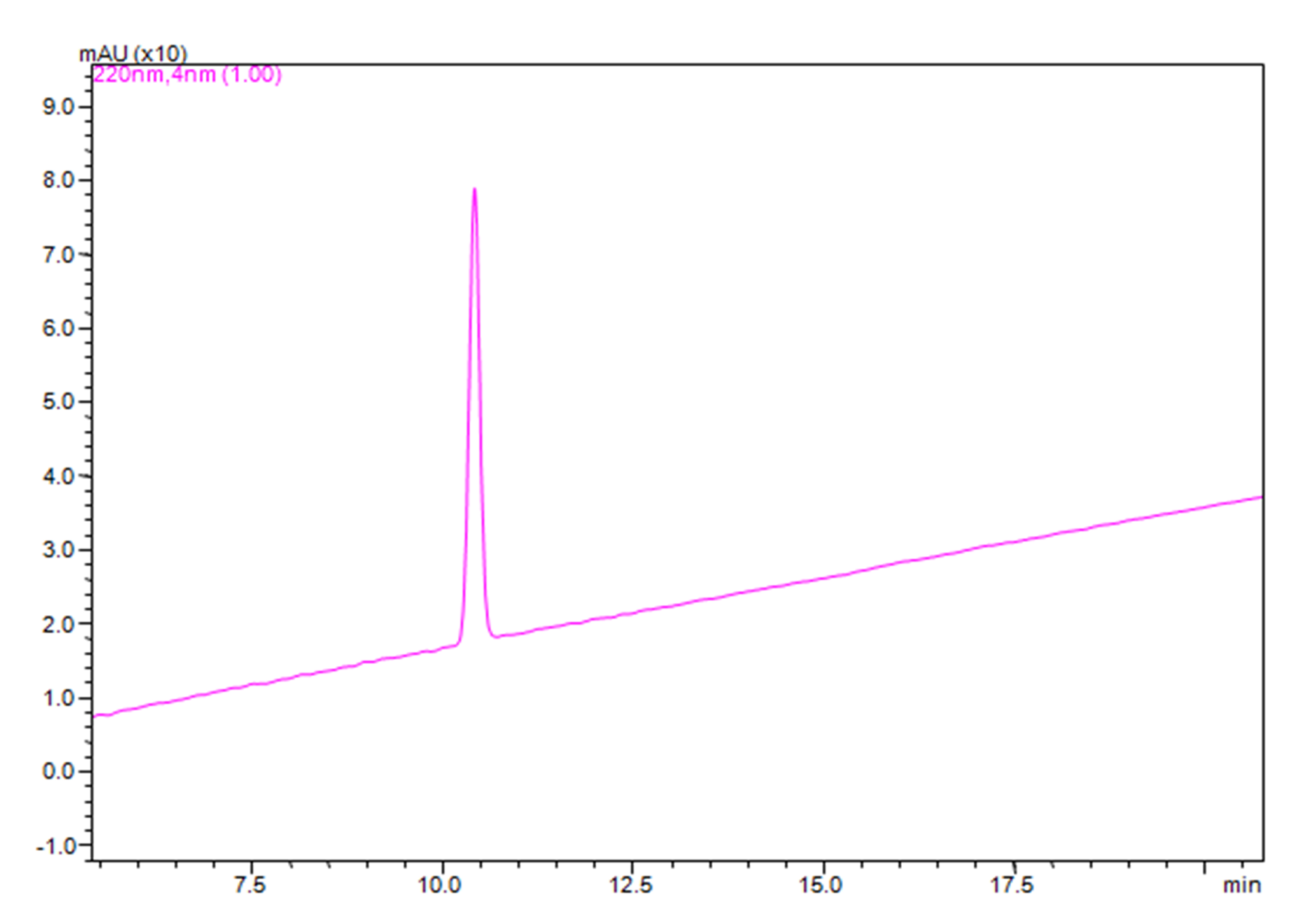

Supplement: Figure S5 — UV chromatogram of 17- O -acetylacuminolide (2). (TIF) [file pone.0015105.s005.tif]

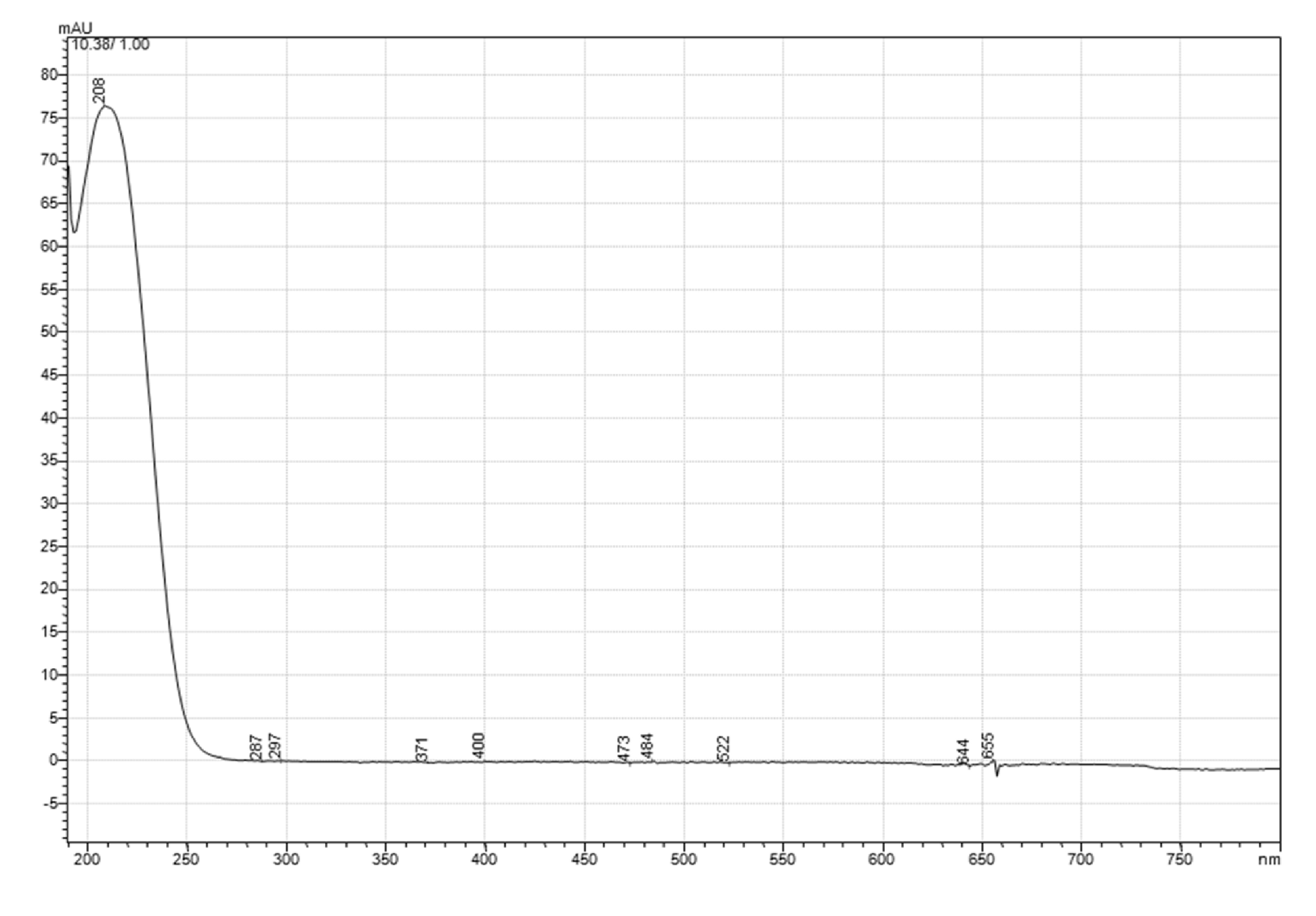

Supplement: Figure S6 — Extracted UV Spectrum of 17- O -acetylacuminolide (2). (TIF) [file pone.0015105.s006.tif]
